# Acute renal failure and rhabdomyolysis in a patient with infectious mononucleosis: a case report

**DOI:** 10.1186/1757-1626-1-222

**Published:** 2008-10-07

**Authors:** Stavros Aloizos, Stavros Gourgiotis, Konstantinos Oikonomou, Paraskevi Stakia

**Affiliations:** 1 Intensive Care Unit, 401 General Army Hospital of Athens, 14 Acheon Street, 15343, Ag. Paraskevi, Athens, Greece; 2 Second Surgical Department, 401 General Army Hospital of Athens, 41 Zakinthinou Street, 15669, Papagou, Athens, Greece; 3 Oncology Department, 401 General Army Hospital of Athens, 23 Filias Street, 15123 Marousi, Athens, Greece; 4 8 Pirgou Street, 16675, Glyfada, Athens, Greece

## Abstract

We report a very rare case of acute renal failure and rhabdomyolysis in an Intensive Care treated 20-years-old male with upper airway obstruction due to Epstein-Barr infection.

In our opinion this was a manifestation of the very rare and potentially lethal propofol infusion syndrome and not a direct complication of the underlying infection, although renal biopsy was not performed in our patient.

## Case presentation

A 20-years-old male with two-week history of tonsillitis, high fever and cervical lymphadenopathy presented to our hospital and was admitted for further management. He had neither past nor family medical history. He had already received broad spectrum antibiotics and corticosteroids to control pharyngeal oedema. He was diagnosed with Epstein-Barr (EBV) infection with an increase in IgM antibodies.

One week later he presented central cyanosis and wheezing and was transferred to the Intensive Care Unit (ICU) due to upper respiratory obstruction where he was intubated.

For sedation and analgesia he was initially administered remifentanyl and propofol at 15 ml/hr (2.14 mg/kg/hr) which was increased gradually at 40 ml/hr (5.71 mg/kg/hr) by the 4^th ^day. He was also administered a triple antibiotic regiment of meropemen, gentamycin and teicoplanin. A tracheotomy was performed for a safer weaning and to better control the pharyngeal oedema.

On the 4^th ^day the patient presented rhabdomyolysis with creatine phosphokinase (CPK) levels > 7000 iu/lt. He was initially managed with hydration and IV bicarbonate administration. Propofol infusion syndrome (PRIS) was suspected, although it was first described in children and without the rest signs and symptoms of lactic acidosis and heart failure that accompany it. He was constantly monitored haemodynamically with a cardiac index of 3.2 to 3.7. Propofol infusion was suspended and midazolam was instead administered for sedation.

The following day the patient presented acute renal failure with oliguria that gradually progressed to complete anuria with creatinine levels reaching a maximum of 7 mg/dl.

From the seventh to the 19^th ^day he was placed on both classic and continuous veno-venous haemodiafiltration (CVVHDF) dialysis for a total of 13 times. At that point the patient progressed to polyuria before finally his renal function returned to normal rate.

Blood, urine and broncho alveolar lavage (BAL) cultures were negative. Bed side abdominal ultrasonography (US) revealed mild liver and spleen enlargement. Abdominal computed tomography (CT) findings were also consistent with the EBV infection.

On the 26^th ^day the patient underwent successful weaning from the ventilator and his tracheal tube was removed. He was transferred back to the Internal Medicine department from where he was discharged in good health a week later.

## Discussion

PRIS is a very rare syndrome with 61 published cases in the English literature. It is mainly observed in male patients (98%) that are administered propofol in very high dosages. In the literature is stated that the propofol administration threshold above which the syndrome may appear is 83 mcg/kg/min (4.98 mg/kg/h) for a period of more than 24 hours, or by others 5 mg/kg/h for more than 48 hours [1].

The threshold was exceeded in our patient (5.71 mg/kg/h) but for a period of less than 24 hours (exactly 18 hours) before the symptoms appeared. The syndrome is characterized by lactic acidosis, rhabdomyolysis, cardiac arrhythmias, liver enlargement, renal failure, heart failure and could lead to death [1].

An odds ratio of 1.93 for every 1 mg/kg/h increase over 5 mg/kg/h has been reported [1].

The underlying mechanism leading to the syndrome is not yet understood but recent studies have focused on injury of the mitochondrial respiratory chain with a subsequent failure of the lipid beta acidosis [1,2].

In our case, we only observed rhabdomyolysis and renal failure without any lactic acidosis or circulatory symptoms, probably because of the high rate of suspicion that led to the immediate interruption of the propofol infusion [3].

Another cause for rhabdomyolysis and acute renal failure in our patient could be the EBV infection by itself. In the English literature there have been a number of cases reported of renal failure in EBV infection primary or secondary due to rhabdomyolysis and the subsequent acute tubular necrosis because of the released myoglobin [4].

The virus has been implicated in intratubular renal disease since his genome has been detected in kidney biopsies. It has also been documented that after the initial infection of the kidneys by the virus, follows a phase of fibration that could lead to chronic renal failure [2].

A kidney biopsy was not deemed of any therapeutic use under the circumstances and was not performed.

If the patient's renal failure was secondary and a direct result of the rhabdomyolysis in our opinion should have responded in the treatment aiming at preventing the renal failure taking also into account his less than extreme CPK levels.

Patients treated in the ICU with much higher levels of CPK have responded much better to the same preventive treatment in accordance with international literature.

Renal failure due to EBV is extremely rare (less than 1:14,000,000) and usually presents as an IgA renal disease, glomerulonephritis, minimal change disease and hemolytic uremic syndrome [2,4].

In conclusions, PRIS is a very rare and potentially lethal syndrome that should be dealt with immediately while EBV infection can extremely rarely be the cause of acute renal failure non responsive to corticosteroid therapy.

In our opinion the renal failure was a manifestation of this syndrome and not a direct complication of the underlying infection, although renal biopsy was not performed in our patient.

## Competing interests

The authors declare that they have no competing interests.

## Authors' contributions

PS and KO analyzed and interpreted the patient data. SA was the responsible doctor of the patient. SG was a major contributor in writing the manuscript. All authors read and approved the final manuscript.

## Consent

Written informed consent was obtained from the patient for publication of this case report and accompanying images. A copy of the written consent is available for review by the Editor-in-Chief of this journal.

## References

[B1] Shimony A, Almog Y, Zahger D (2008). Propofol infusion syndrome: a rare cause of multi-organ failure in a man with complicated myocardial infarction. Isr Med Assoc J.

[B2] Nadasdy T, Park CS, Peiper SC, Wenzl JE, Oates J, SiIva FG (1992). Epstein-Barr virus infection-associated renal disease: diagnostic use of molecular hybridization technology in patients with negative serology. J Am Soc Nephrol.

[B3] Fudickar A, Tonner PH, Mihaljovic Z, Dellien C, Weiler N, Scholz J, Bein B (2008). Suggested beginning of propofol infusion syndrome in an adult patient without lactacidosis: a case report. Eur J Anaesthesiol.

[B4] Friedman BI, Libby R (1986). Epstein-Barr virus infection associated with rhabdomyolysis and acute renal failure. Clin Pediatr (Phila).

